# Effect of Pre-Stressing on the Acid-Stress Response in *Bifidobacterium* Revealed Using Proteomic and Physiological Approaches

**DOI:** 10.1371/journal.pone.0117702

**Published:** 2015-02-17

**Authors:** Junhua Jin, Qian Qin, Huiyuan Guo, Songling Liu, Shaoyang Ge, Hongxing Zhang, Jianyun Cui, Fazheng Ren

**Affiliations:** 1 Food Science and Engineering College, Beijing University of Agriculture, Beijing Laboratory for Food Quality and Safety, Beijing, 102206, China; 2 College of Food Science and Nutritional Engineering, China Agriculture University, Beijing, 100083, China; 3 Beijing Higher Institution Engineering Research Center of Animal Product, Beijing, 100083, China; 4 Key Laboratory for Functional Dairy, China Agriculture University, Beijing, 100083, China; 5 Beijing Resources Yatai Feed Technology Co., LTD, Beijing, China; Pacific Northwest National Laboratory, UNITED STATES

## Abstract

Weak acid resistance limits the application of *Bifidobacteria* as a probiotic in food. The acid tolerance response (ATR), caused by pre-stressing cells at a sublethal pH, could improve the acid resistance of *Bifidobacteria* to subsequent acid stress. In this study, we used *Bifidobacterium longum* sub. *longum* BBMN68 to investigate the effect of the ATR on the acid stress response (ASR), and compared the difference between the ATR and the ASR by analyzing the two-dimensional-PAGE protein profiles and performing physiological tests. The results revealed that a greater abundance of proteins involved in carbohydrate metabolism and protein protection was present after the ASR than after the ATR in *Bifidobacterium*. Pre-stressing cells increased the abundance of proteins involved in energy production, amino acid metabolism, and peptidoglycan synthesis during the ASR of *Bifidobacterium*. Moreover, after the ASR, the content of ATP, NH_3_, thiols, and peptidoglycan, the activity of H^+^-ATPase, and the maintenance of the intracellular pH in the pre-stressed *Bifidobacterium* cells was significantly higher than in the uninduced cells. These results provide the first explanation as to why the resistance of *Bifidobacterium* to acid stress improved after pre-stressing.

## Introduction

Several species in the genus *Bifidobacterium* are considered to be probiotic [1]. This has led to their widespread application in probiotic products. However, the acid resistance of *Bifidobacterium* is generally weak. Acid stress therefore represents a major challenge to this organism, reducing viability and its putative probiotic effects [2, 3]. Strengthening acid tolerance is critical to enhance the survival of *Bifidobacterium*, and therefore to ensure the quality and functionality of probiotic products.

Several studies have suggested that the acid tolerance response (ATR) pre-induced at sublethal pH could strengthen the acid resistance of cells to subsequent lethal pH, known as the acid stress response (ASR). In *Bifidobacteria*, the response can by no means be described as robust as found in other gram positive or negative species, however, ATR in *Bifidobacteria* provided a good model for investigating the mechanism of response to low pH.Previous studies demonstrated that the mechanism involved in the ATR in *Bifidobacterium* involves the discharge of H^+^ by H^+^-ATPase, the blocking of H^+^ by the cell membrane and cell wall, the neutralization of H^+^ by alkalinity products, intercellular communication via quorum sensing, and other signal transmission systems [4–7]. The mechanism of ATR was studied by exploring changes in the physiological status of cells when they were pre-stressed at sublethal pH; however, what happens to the ASR after the cells are pre-stressed, and the differences between the responses to sublethal and lethal pH in *Bifidobacterium* remain unknown.

*Bifidobacterium longum* subsp. *longum* BBMN68, which was isolated from centenarians in Bama village, China, exhibited many probiotic functions. However, the weak acid resistance of *B*. *longum*BBMN68 reduced its probiotic effects. In this study, we investigated the effect of the ATR on the ASR in *B*. *longum* BBMN68. We analyzed changes in the protein profile and physiology of this organism after pre-stressing the cells. In this study, we discovered that pre- stressing increased the abundance of proteins involved in energy production, amino acid metabolism, and peptidoglycan synthesis during the ASR of *B*.*longum*BBMN68. Moreover, after ASR, the content of ATP, NH_3_, thiols, and peptidoglycan, and the activity of H^+^-ATPase and maintaining intracellular pH in the pre- stressed *Bifidobacterium* was significantly higher than that in uninduced cells. Our findings provide insight into the mechanism behind the ASR in *Bifidobacterium* and how it is affected by induction of the ATR.

## Materials and Methods

### Strain and culture conditions

*Bifidobacterium longum* subsp. *longum* BBMN68 (CGMCC 2265,China General Microbiological Culture Collection Center) was cultured in modified MRS medium (supplemented with 0.05% L- cysteine HCl)at an initial pH of 6.5 at 37°C under an aerobic conditions. The pre- stressing and stress pHs were measured as described previously(Jin et al. 2012). ASR was stimulated by culturing at pH3.5 for 2 h, whereas ATR was caused by culturing at pH4.5 for 2 h.

**Fig 1:**
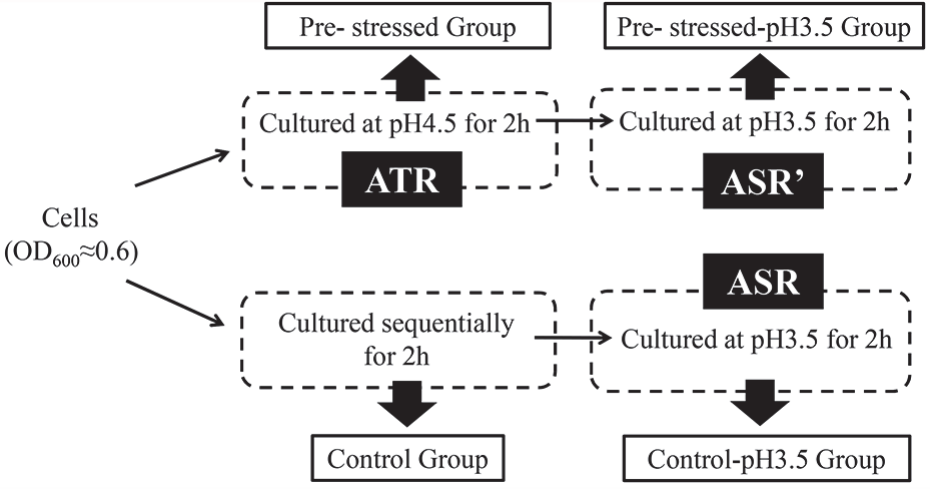
Schematic of the experimental design. Cultures in the mid-exponential phase (OD_600_ = 0.6) were harvested and divided into two. Half of the cells were cultured for 2 h in the original medium, serving as the control group, and the other half of the cells wereculturedat pH 4.5 for 2 h, serving as the pre-stressed group. Then, the cells from both groups were harvested and challengedat a lethal pH 3.5 for 2 h. The resulting cells served as the control-pH 3.5 group and the pre-stressed-pH 3.5 group. ATR, response to pH 4.5; ASR, response to pH 3.5 of the cells in the control group; ASR, response to pH 3.5 of the cells in the pre-stressed group.

Cultures in the mid-exponential phase (OD_600_ = 0.6) were harvested and divided into two parts. The first part cells were cultured for 2 h in the original medium, serving as control group, and the second part cells were adapted at pH4.5 for 2 h, serving as pre-stressed group. Then the cells harvested from control group and pre-stressed group were stress in MRS at lethal pH3.5 for 2 hours, respectively. The resulted cells served as control-pH3.5 group and pre-stressed- pH3.5 group, respectively (As shown in Fig. 1). One hundred milliliter BBMN68 cells were harvested under different conditions in a repeat experiment.

### 2D-PAGE analysis of protein profiles

Two dimensional polyacrylamide gel electrophoresis (2D-PAGE) analyses were performed as described previously [8], with minor modifications.

**Whole-cell protein extraction**. The cells from 100 mL cultures were harvested by centrifugation at 8,000 × *g* at4°Cfor 5 min. The pellets were then washed twice with pre-cooled PBS, and once with pre-cooled sterile ultrapure water. The pellet (∼1.0 g) was resuspended in 3 mL lysis buffer containing 7 M urea,2 M thiourea, 4% (w/v) CHAPS (GE Healthcare), 65 mM dithiothreitol(DTT) (Sigma), 2% (v/v) pH 4–7 immobilized pH gradient (IPG) buffer (GE Healthcare), and 240 μL protease inhibitor cocktail (GE Healthcare, 1 cocktail pill was dissolved in 2 mLultrapure water). The cell suspension was sonicated for 45 min on ice using an ultrasonic processor (Ningbo ScientzBiotechnology Co., Scientz-IID) at 300 W with 3 s pulses at 5 s intervals. Next, 30 μL of nuclease mix (GE Healthcare) was added to the cell lysate, and the mixture was incubated for 1h at 25 °C, and then centrifuged for 15 min at 20,000 × *g* at 4°C. The supernatant was collected, and the protein concentration was quantified using the Bradford method [9]. Three independent gels (IPG strips; technical replicates) were run for each sample in 2D-PAGE, as described below.

**2D-PAGEand image analysis.** For electrophoresis in the first dimension, total whole cell protein(800 mg) was loaded onto IPG strips (18 cm, pH 4–7; GEHealthcare) that had been rehydrated overnight in 350 μL rehydration solution containing 8 M urea, 4% (w/v) CHAPS, 18mM DTT, 2% (v/v) pH 4–7 IPG buffer, and 0.02% bromophenol blue. Isoelectric focusing(IEF) was performed in an IPG phor system(GE Healthcare), with the following voltage gradient: 200 V for 800 V^.^ h, 500 V for 1000 V^.^ h, an increase from 500 to 1000 V for 800 V^.^ h, 1000 V for 1000 V^.^ h, an increase from 1000 to 8000 V for 13500 V^.^ h, and 8000 V for 80000 V^.^ h, for a total of 97.1kVh. The strips were equilibrated in equilibration buffer (50 mM Tris/HCl (pH 8.8), 6 M urea, 30% (v/v) glycerol, 2% (w/v)sodium dodecyl sulfate(SDS), and 0.002% (w/v)bromophenolblue): first in buffer containing 1% (w/v) DTT for 15 min, and then containing2.5% (w/v) iodoacetamide for 15 min. For electrophoresis in thesecond dimension, SDS-PAGE (12.5%) was performed using an EttanDalt Twelve (GE Healthcare) at 10 mA for 1 h, then at 40 mA per gel until the dye front reached the bottom of the gel. Proteins were then stained using a sensitive colloidal Coomassie Brilliant BlueG250 [10]. The gels were scanned at 600d.p.i. (Perfection 4900 scanner, Epson).Image analysis was performed using ImageMaster 2D Platinum version 5.0 software (GE Healthcare), which automatically found protein spots within the image and quantify their densities on the basis of percent volume (values were calculated by integrating the spot optical intensity over the spot area). Signals from non-protein sources were filtered out, and the protein spots were normalized before pairwise image comparisons were performed. All protein spots exhibiting at least 2-fold differences between the different groups were evaluated for statistical significance using the Student’s t-test. All spots with *p* values of <0.05 were inspected to ensure good spot assignment and volume integration. Three biological repeats were performed in each group, and three technical repeats were performed in each biological repeat experiment.

**In-gel digestion and protein identification.** Protein spots of interest were manually excised and collected from all replicate gels as described previously [8]. Peptides from in-gel-digested proteins were then identified using4800 Plus MALDI-TOF/TOF (AB SCIEX, US), which was performed by Beijing Biomes Sci-tech Co.,Ltd.

### Physiological experiments

The intracellular pH (pH_in_), ATP content, NH_3_ content, peptidoglycan content, and H^+^-ATPase activity were assessed as described below; all methods were modified from previous studies[11,12,13,14].The detail of physiological test methods was described in S1 File. Six biological repeats were performed in each experiment.

**Determining intracellular pH (pH**_**in**_ **)** Intracellular pH was determined using a modified version of the method described by Bracey [11]. The pellet from a bacterial culture was diluted to an OD_600_ of ∼0.8, washed in sterile water, and centrifuged at 8,000 × *g*for 10 min. The pellet was then resuspended in an equal volume of 100 mM citric/phosphate buffer at pH 4.0, before 5(6)-carboxyfluorescein diacetate (cFDA) was added to a final concentration of 10 μM, and the cells were incubated at 37°C for 60 min to load the cells with cFDA in an anaerobic culture box. The loaded cells were then harvested by centrifugation at 4,000 rpm for 10 min, and then resuspended in a double volume of 100 mM citric/phosphate buffer for washing. The harvested cells were resuspended in an equal volume of 100 mM citric/phosphate buffer at a specified pH (6.2, 5.0, 4.5, 4.2, 4.0, 3.5, and 3.0). The total fluorescence was measured using a Shimadzu RF-5301 fluorometer (Shimadzu UK, Haverhill, Suffolk) with excitation at 400 and 500 nm, and emission at 525 nm (bandwidth 10 nm). The background fluorescence of the culture supernatant (after cells had been removed by centrifugation at 13,500 rpm for 4 min) alone was subtracted from the total fluorescence. The intracellular pH was then determined based by plotting the fluorescence intensity of cFDA (495/435 nm) against the calibration curve.

To plot the calibration curve, a final concentration of 10μMcFDA was added to 100 mM citric/phosphate buffer as well as permeabilized cells of pHs from pH4.0 to pH7.0 (increments of 0.2 of a pH unit). To permeabilized the cells, cultures were diluted to an OD_600_ of ∼0.8, and were then exposed to 5.0 mM amphotericin B for 60 min at 37°C. The fluorescence was measured as described above, and the calibration curve for fluorescence intensity (495/435 nm) was plotted against pH (results not shown).

**ATP determination** One hundred milliliter cultures were centrifuged at 6000 × *g* for 10 min at 4°C, and resuspended in 0.75 mL of pre-cooled 5.6% (v/v) perchloric acid. The cells were sonicated rapidly in an ice-bath, and then centrifuged at 3,500 × *g* for 1 min at 4°C. The supernatant was added to an equal volume of 1 M dipotassium phosphate (pH 6.5), and then centrifuged again at 3,500 × *g* for 1 min at 4°C. The supernatant was filtered using a 0.22μm membrane. The processing was finished in an ice bath. The supernatant was analyzed using HPLC with the following conditions: an ODS HYPERSIL C18 column; a mobile phase of 50 mM potassium phosphate (pH6.5) with a flow rate of 1.0 mL min^-1^; temperature, 25°C; UV absorbance of 254 nm. An ATP standard (Sigma) curve was calculated using the same HPLC method. The cellular ATP content was calculated using the standard curve.

**Measuring ATPase activity** One hundred milliliter cultures were collected from the different treatment groups, and H^+^-ATPase extraction and calculation were completed as described previously [12].

**Determining NH**_**3**_ **content** Fifty-milliliter BBMN68 cultures from the different test groups described above were collected by centrifugation. The precipitates were washed once, and then sonicated in 5 mL of 0.1M hydrochloric acid solution containing 0.2% TDPA in an ice bath. They were then centrifuged at 5,000 × *g* for 20 min, and the supernatants were deproteinized by mixing with an equal volume of 40% (w/v) trichloroacetic acid, and incubated on ice for 10 min. After centrifugation at 15,000 × *g* for 15 min, a 100-μL aliquot of the supernatant was dried in a vacuum concentrator (Speed-Vac), and then re-dissolved in 100 μL of reaction buffer. Derivatization and NH_3_-determination of the samples were then performed immediately as described previously [13].

**Determination of peptidoglycan content** One hundred milliliter cultures from the different test groups were collected by centrifugation at 8,000 × *g* for 10 min. The pellets were added to an equal volume of boiling 8% (w/v) SDS, incubated with agitation for 30 min, cooled, and then centrifuged at 16,000× *g* for 20min. The pellets were washed with equal volumes of 2% (w/v) SDS, 0.5 M NaCl, and 0.85% (w/v) NaCl. The pellets were dried to a constant weight, and then resuspended in 50mL 10 mM Tris-HCl (pH8.0) containing 100μg/mL bovine pancreatic trypsin (Kayon, Shanghai). The mixtures were stirred at 20°C for 20 min, and then centrifuged at 73,000 × *g* for 1 h. The gelatinous material that sedimented was resuspended in 50 mL of 10 mMTrisHCl (pH8.0) containing 5 mM MgCl_2_, 0.1 μL/mL deoxyribonuclease I(70U/μL, TaKaRa),and 0.1 μL/mL ribonuclease(100mg/mL, Tiangen). The suspension was incubated at 20°C for 20 min, and then centrifuged at 73,000 × *g* for 1 h. The pellets were washed three times by suspending in distilled water and centrifuging. The cell envelope extracts that were enriched in peptidoglycan sacculi were recovered, and then lyophilized. Lyophilized 10–20 mg samples were added to 10 mL of 6 M HCl for hydrolysis at 105°C for 4h, and then cooled. Next, 6 M NaOH was added to neutralize the solution at a pH of 7.0–8.0, and distilled water was added to a final volume of 25 mL. The N-acetylglucosamine content was then determined using a modified Morgan-Elson method described previously. Three replicates were performed for each treatment, unless otherwise stated.

## Results

### Proteomic analysis

**Fig 2:**
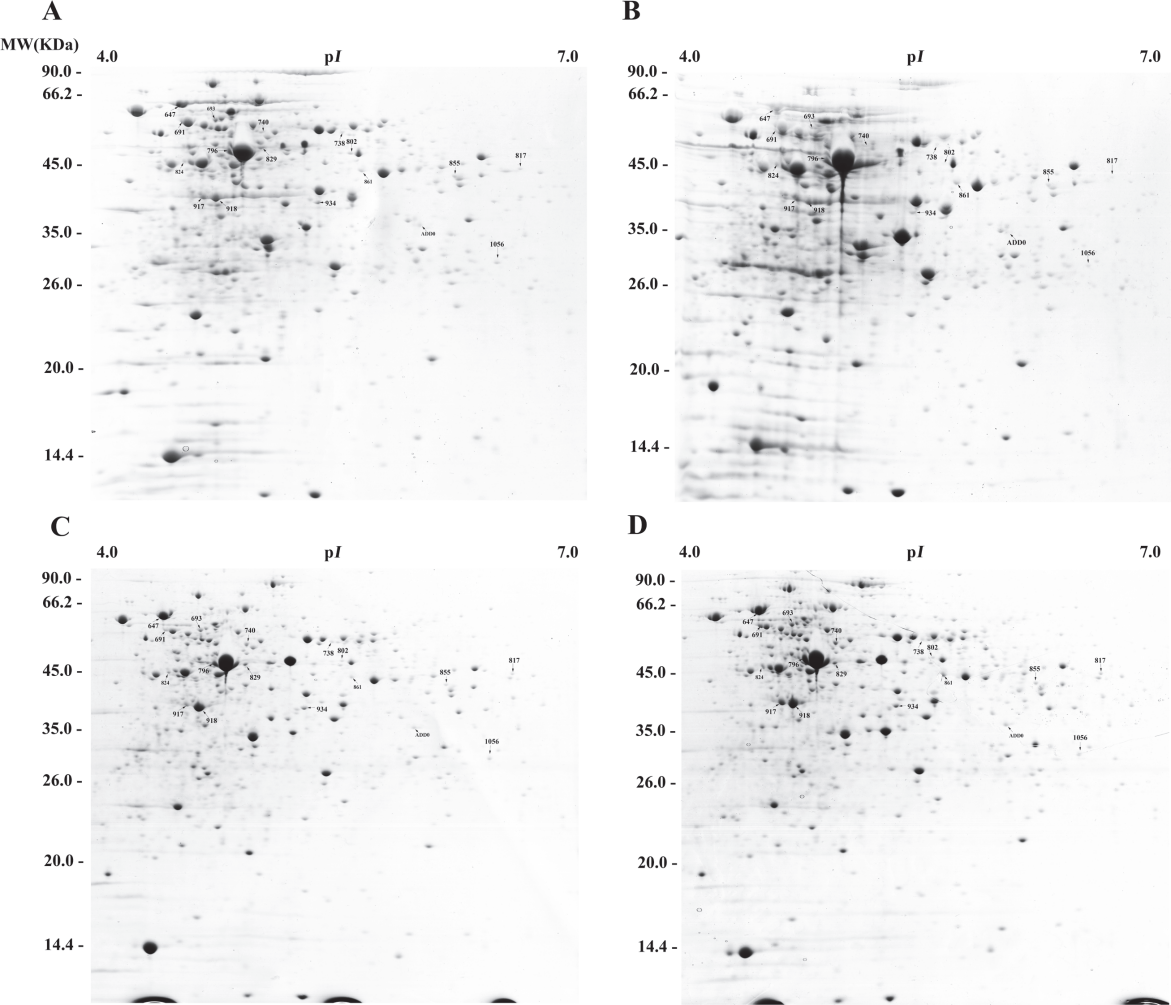
Protein-profiles of Bifidobacteriumlongum subsp. longum BBMN68 cells belonging to the different groups. A, control group; B, pre-stressed group; C, control-pH 3.5 group; D, pre-stressed-pH 3.5 group. Numbers indicate the protein spots of interest, for which information is listed in Table A in S1 File.

**Fig 3:**
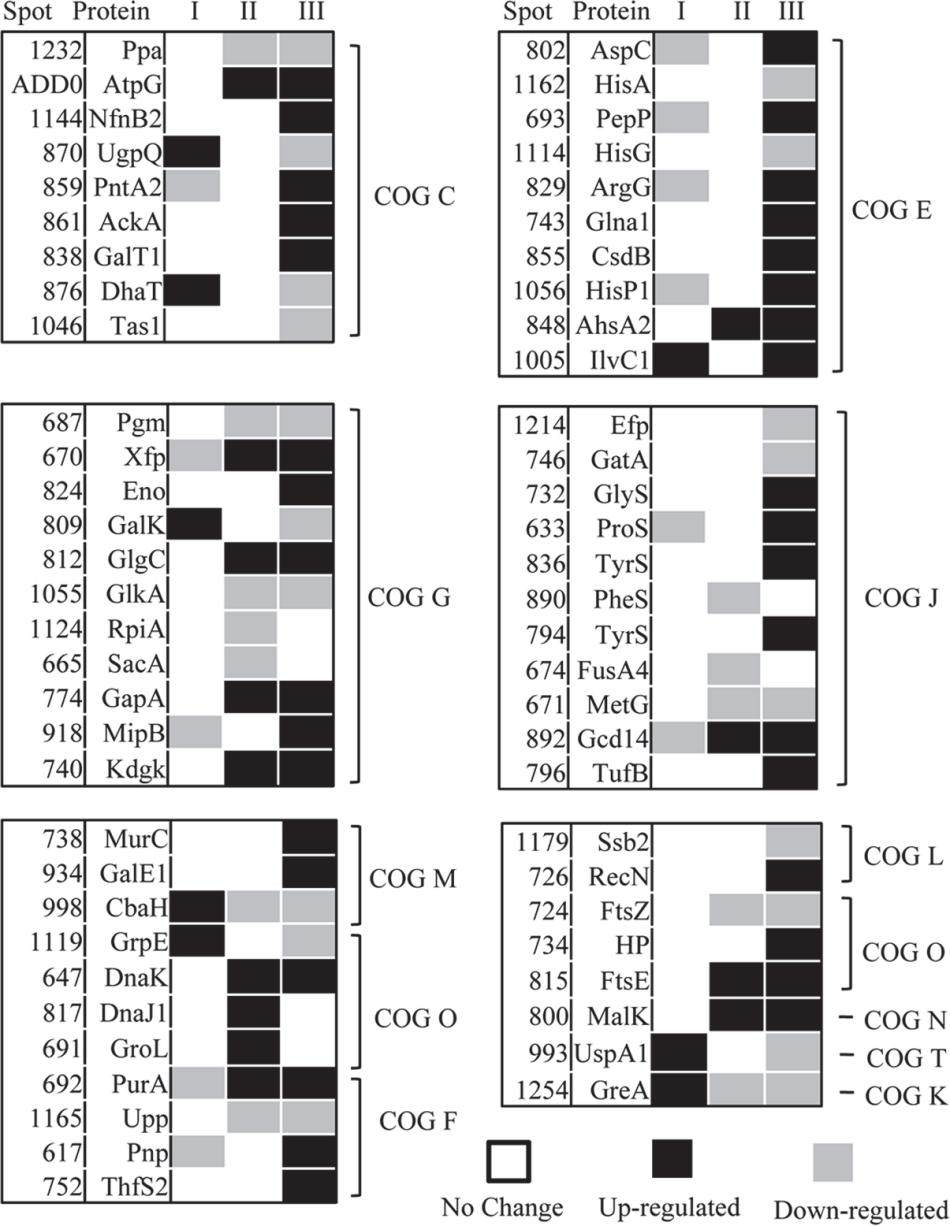
Changes in the protein profile during the response to an acid environment in *Bifidobacterium*. Line I, the value = the abundance of protein in the pre-stressed group were normalized against the control group cells; Line II, the value = the abundance of protein in the control-pH 3.5 group were normalized against the control group cells. Line III, the value = = the abundance of protein in the pre-stressed-pH3.5 group were normalized against the pre-stressed group cells. Black, up-regulated by ≥ two-fold; gray, down-regulated ≥ two-fold; white, no significant change. The solid numbers in the corresponding lines I, II, and III are listed in the columns ATR*,ASR**,and ASR***, respectively, in Table A in S1 File.

A previous study suggested that pre-stressing at pH 4.5 for 2 h could increase the survival of *B*. *longum*BBMN68 at pH 3.5 by 60-fold [5]. Therefore, in the current study, we analyzed the difference between the response to sublethal pH 4.5 and lethal pH 3.5 at the proteome level (the difference between ATR and ASR is described in Fig. 1). We also investigated the effect of pre-stressing at pH 4.5 on the response to lethal pH 3.5 by comparing the protein profiles and physiological parameters of *Bifidobacterium*(the difference between ATR and ASR is described in Fig. 1). The proteins whose abundance changed are shown in Fig. 2 and Fig. 3 and Table A in S1 File.

### Comparison between the protein profiles of *B*. *longum* BBMN68 in the pre-stressed group and the control group

To investigate the effect of pre-stressing on the abundance of proteins in *B*. *longum*BBMN68, we assessed the changes in protein profiles in the pre-stressed and control groups using 2D-PAGE (Fig. 2). The abundance of 20 proteins changed after pre-stressing at pH 4.5, eight of which were upregulated by more than two-fold (Fig. 3-I, Table A in S1 File). The abundant upregulated proteins included the molecular chaperone (GrpE), an enzyme involved in the synthesis of isoleucine and valine (IlvC1), glucokinase (GalK), and conjugated bile acid hydrolase (CbaH). Twelve proteins were downregulated by more than two-fold, including phosphoketolase (Xfp), transaldolase (MipB), and an ABC-type amino acid transport system ATPase component (HisP1).

### Comparison between the protein profiles of *B*. *longum* BBMN68 in the control- pH3.5 group and the control group

The 2D-PAGE data also revealed changes in the protein profile of *B*. *longum*BBMN68 that had encountered an acid stress environment. The abundance of 25 proteins changed in *B*. *longum*BBMN68 after acid stress at pH 3.5 for 2 h (Fig. 3-II, Table A in S1 File). Thirteen proteins were upregulated by more than two-fold, including phosphoketolase (Xfp), fructokinase (KdgK), ABC-type sugar transport systems, ATPase components (MalK), and molecular chaperones (DnaK, DnaJ1, and GroEL). In addition, abundance of 12 proteins were downregulated, including enzymes involved in glycometabolism (Pgm, SacA, GlkA, and RpiA), transcription elongation factor (GreA), translation elongation factor (FusA4), CbaH, methionyl-tRNA synthetase (MetG), and prolyl-tRNA synthetase (ProS).

### Comparison between the protein profiles of *B*. *longum*BBMN68 in the pre-stressed-pH 3.5 group and the pre-stressed group

Data revealed that the abundance of 53 proteins differed significantly between the pre-stressed-pH 3.5 group and the pre-stressed group (Fig. 3-III, Table A in S1 File). Thirty-four of these proteins were upregulated by more than two-fold, including enzymes involved in glycolysis (AckA, Xfp, MipB, Eno, GapA, and KdgK), ketol-acid reductoisomerase (IlvC1), UDP-N-acetylmuramate-L-alanine ligase (MurC), and a molecular chaperone (DnaK). In addition, abundance of 19 proteins were downregulated by more than two-fold, including glucokinase (GlkA), GalK, phosphoglucomutase (Pgm), enzymes involved in histidine metabolism (HisA and HisG), translation elongation factor (Efp), GreA, CbaH, a molecular chaperone (GrpE), and a universal stress protein (UspA1).

### Physiological analysis

**Fig 4:**
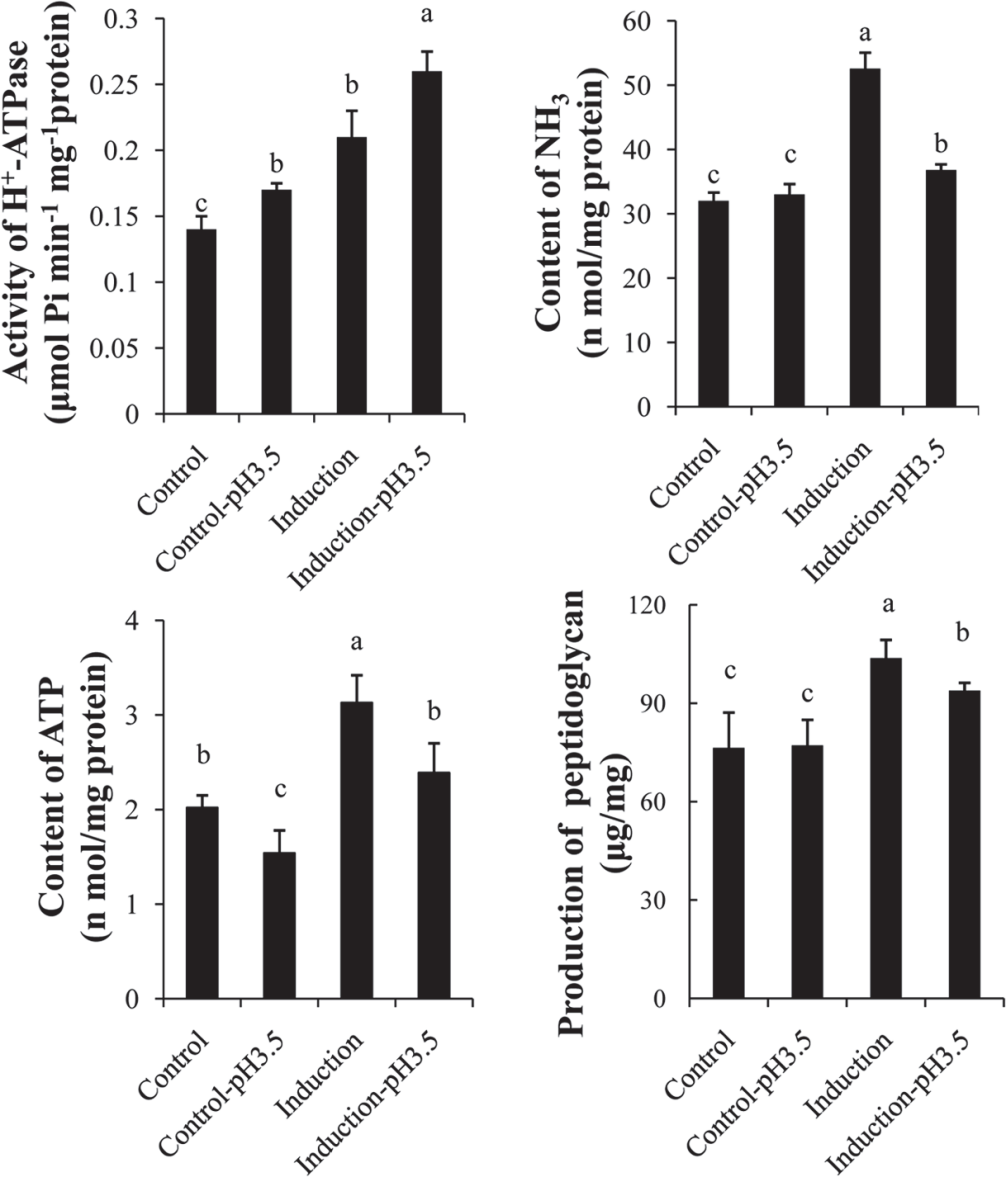
Comparison of the physiological characteristics of *Bifidobacterium* in the different treatment groups. The same letters indicate no significant difference among the detected values (*p* > 0.05).Biological repeats, n = 6.

**DeterminingATP content**. To assess the relationship between the energy requirements of *Bifidobacterium* and their response to an acid environment, we next measured the ATP content of the cells in different groups. As shown in Fig. 4, the ATP content in the BBMN68 control group was 2.02 nmol/mg protein, which was significantly lower than the pre-stressed group for which 3.28 nmol/mg protein was observed. This indicated that pre-stressing improved the ATP-producing ability of *Bifidobacterium*. After acid stress at pH 3.5, the ATP content decreased significantly, without or with pre-stressing, to 1.54 and 2.39 nmol/mg protein, respectively. Significant differences were detected in both groups.

**H** ^**+**^ **-ATPase activity.** H^+^-ATPase plays an important role in the ASR in bacteria [15, 16]. We next investigated the effect of different treatments on the H^+^-ATPase activity of the different groups of *B*. *longum*BBMN68. As shown in Fig. 4, the H^+^-ATPase activity in the pre-stressed group was 0.21 μmol Pi/min/mg protein, which was significantly higher than in the control group (0.14 μmol Pi/min/mg protein). After incubation at pH 3.5 for 2 h, the H^+^-ATPase activity in both groups of *B*. *longum*BBMN68 with or without pre-stressing increased significantly. However, *B*. *longum*BBMN68 cells in the pre-stressed-pH 3.5 group showed higher H^+^-ATPase activity compared with the control-pH 3.5 group.

**Determining the NH**_**3**_ **cellular content.** Neutralizing intracellular H^+^ is an important component of the ASR. The protein profiles suggested that the ability of amino acid metabolism, which is the major source of alkaline metabolites, was altered during the ASR and the ATR in *Bifidobacterium*. Therefore, we determined the NH_3_ content of *Bifidobacterium* from typical alkaline metabolites under different conditions. The data suggested that the NH_3_ content of *B*. *longum*BBMN68 in the control group was 32 nmol/mg protein, which was significantly lower than in the pre-stressed group, which gave 53 nmol/mg protein. After acid stress at pH 3.5, there was no significant change in the NH_3_ content in *B*. *longum*BBMN68 in the control group; however, the NH_3_ content decreased significantly in the *B*. *longum*BBMN68 pre-stressed group to 37 nmol/mg protein (Fig. 4).

**Determining peptidoglycan production.** Peptidoglycan contributes to the integrity and intensity of the cell walls of Gram-positive bacteria [17, 18]. Because of the protein profile results, which suggested that peptidoglycan synthesis was affected in the ATR and the ASR, we assessed peptidoglycan production in cells from the different treatment groups. As shown in Fig. 4, peptidoglycan production was 76 μg/mg of dry cell weight and there was no significant change after acid stress at pH 3.5 for 2 h. However, there was a significant increase in peptidoglycan production after pre-stressing at pH 4.5 for 2 h to 103 μg/mg of dry cell weight. Peptidoglycan production in cells in the pre-stressed-pH 3.5 group was significantly higher than in the control-pH 3.5 group. These results suggested that the cell wall in the induced cells had improved integrity and intensity.

**Fig 5:**
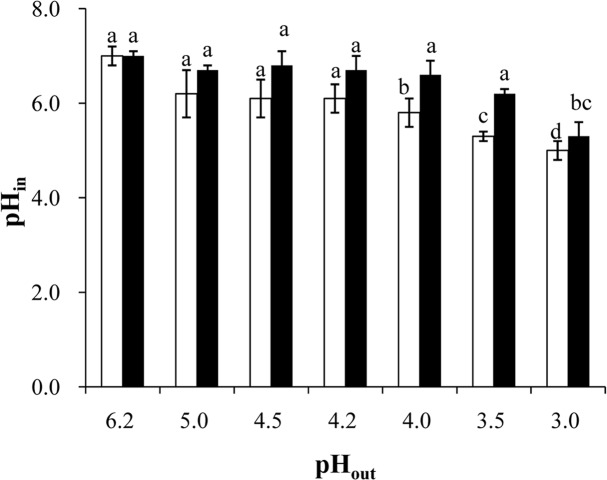
Effect of environmental pH on the intracellular pH in *Bifidobacterium*. White bars, the control cells; black bars, the pre-stressed cells. The same letters indicate no significant difference among the pH_in_ values in *B*. *longum*BBMN68 cells under different environmental conditions (*p*> 0.05). Biological repeats, n = 6.

**Determining the intracellular pH (pH**_**in**_ **).** To investigate the effect of pre-stressing at pH 4.5 on the ability of *B*. *longum*BBMN68 to maintain pH balance, we detected the pH_in_ of the cells with or without pre-stressing in different pH environments. As shown in Fig. 5, the pH_in_ was 7.0 in a pH 6.2 environment in both groups. In pH 4.0–5.0 environments, the cells without pre-stressing had a lower pH_in_ than cells with pre-stressing; however, no significant difference was detected between the two groups. In a pH 3.5 environment, the pH_in_ was 5.3 in *B*. *longum* BBMN68 cells without pre-stressing, which was significantly lower than the pH 6.2 observed in *B*. *longum*BBMN68 cells that were pre-stressed. In a pH 3.0 environment, the pH_in_ decreased to 5.0 in both groups of cells.

## Discussion

In a previous study, we analyzed the mechanism involved in the ATR in *Bifidobacterium* at the transcriptome level using RNA-seq [5]. In this study, we furthered our analysis by investigating the ATR in *Bifidobacterium* at the proteome level. However, the expression of genes encoding proteins whose abundance changed by at least two-fold, did not show a similar trend at the transcriptional level during the ATR in *B*. *longum*BBMN68 (as shown in Fig. A in S1 File). This type of phenomenon is common in nature. The absence of an mRNA-protein correlation for the subset of investigated genes suggested that the relationship between mRNA and protein is not strictly linear, but has a more intrinsic and complex dependence, deviating from the classical view referred to as the molecular dogma. Different regulation mechanisms (such as synthesis and degradation rates), acting on both the synthesized mRNA and the synthesized protein, affect the amount of the two molecules differentially.

### Difference between the ATR and the ASR

In *B*. *longum*BBMN68, the proteins that were upregulated after the ATR were different from those upregulated after the ASR. This suggested that the response to a sub-lethal pH environment (ATR) differed from the response to a lethal pH environment (ASR) in *Bifidobacteria*. Faced with a lethal pH environment (pH 3.5), the abundance of the Xfp, GapA, and GlgC proteins, which are all involved in carbohydrate metabolism, were upregulated by more than two-fold in *B*. *longum* BBMN68, whereas no significant changes in protein levels were detected in *B*. *longum* BBMN68 faced with a sublethal pH environment (pH 4.5) (Fig. 3, Table A in S1 File). The abundance of the AckA, MipB, and Eno proteins, which play roles in glycolysis, were also upregulated significantly in *B*. *longum* BBMN68 in the pre-stressed group faced with a lethal pH environment. These results suggested that carbohydrate metabolism was enhanced in the ASR in *Bifidobacterium*. This implies that the requirement for carbohydrate and energy increases in *Bifidobacterium* in a lethal pH environment. This is supported by the data showing the weaker acid tolerance of *B*. *longum*BBMN68 in a lethal pH environment without glucose (data not shown) and the lower ATP content in *B*. *longum*BBMN68 cells in a lethal pH environment (Fig. 4).

When *B*. *longum*BBMN68 was incubated at a lethal pH, the abundance of proteins DnaK, DnaJ1, and GroEL, as well as the thiol content (data not shown), were increased significantly. These molecules and products play roles in protein protection in bacteria in a stressed environment [19, 20]. This suggests that protein structural integrity was threatened at a lethal pH, and therefore the abundance of molecular chaperones was increased to protect proteins against damage in cells in an acid stress environment.

Furthermore, the abundance of the γ-subunit of F_1_F_0_-ATPase was upregulated in *B*. *longum*BBMN68 faced with a lethal pH, which supports the hypothesis that F_1_F_0_-ATPase plays an important role in the ASR in *Bifidobacteria*. This conclusion is consistent with previous studies revealing that F_1_F_0_-ATPase contributes to acid tolerance in bacteria by discharging redundant H^+^ [16].In contrast, the higher activity of F_1_F_0_-ATPase in *B*. *longum*BBMN68 in the pre-stressed-pH 3.5 group compared with the control-pH 3.5 group suggested that pre-stressing at pH 4.5 prior to lethal stress promoted F_1_F_0_-ATPase activity in *Bifidobacterium* in a lethal pH environment. This is a powerful explanation as to why pre-stressing improved the acid tolerance of *Bifidobacteria*.

### Effect of pre-stressing on the ASR in *B*. *longum*BBMN68

In a lethal pH environment (pH 3.5), the abundance of 34 proteins was upregulated in *B*. *longum* BBMN68 in the pre-stressed-pH 3.5 group compared with the pre-stressed group (during the ASR), whereas the abundance of 13 proteins was upregulated in *B*. *longum* BBMN68 in the control-pH 3.5 group compared with the control group (during the ASR), as shown in Fig. 3 and Table A in S1 File. The different protein profiles between the pre-stressed-pH 3.5 group and the control-pH 3.5 group suggested that pre-stressing affected the response of *Bifidobacteria* to a lethal pH environment. Similarly, Sanchez *et al*. (2007) assessed *Bifidobacterium* in a low pH environment and reported that more proteins were upregulated in an acid-resistant mutant compared with the wild-type strain [6]. This result suggested that *Bifidobacterium* cells with increased acid resistance would be able to respond to acid stress more strongly by upregulating the relevant proteins. Hence, pre-stressing cells at pH 4.5 has beneficial effects on the ASR in terms of protein levels in *B*. *longum*BBMN68.

**Effect on energy production.** In a lethal pH environment, KdgK, AckA, MipB, and Eno, proteins which play a role in carbohydrate and energy metabolism (Fig. 3 and Table A in S1 File), were specifically upregulated during the ASR in *B*. *longum* BBMN68 cells that had been pre-stressed. In addition, the abundance of aspartate aminotransferase (AspC), argininosuccinate synthase (ArgG), glutamine synthetase (Glna1), selenocysteinelyase (CsdB), and the ABC-type amino acid transport system ATPase component (HisP1) proteins were all upregulated during the ASR in *B*. *longum*BBMN68. Carbohydrate and amino acid metabolism are the main sources of energy in cells. Therefore, these results suggested that pre-stressing enhanced the activity of carbohydrate and amino acid metabolism to satisfy the energy requirements in a lethal pH environment. Furthermore, the results of physiological tests suggested that pre-stressing enhanced ATP production in *B*. *longum*BBMN68 (Fig. 4). The increased ATP provided *B*. *longum*BBMN68 cells with a higher ability to resist acid stress. This is consistent with the observations of the two-dimensional profile in the current study, and the inference of transcriptome data reported previously [5].

**Effect on the cell wall and cell membrane.** The cell envelope and membrane are the first targets of physicochemical stress. In *Bifidobacteria*, they are mainly composed of lipids, peptidoglycans, and exopolysaccharides. In a lethal pH environment, MurC and GalE1, which are involved in peptidoglycan synthesis, were upregulated during the ASR. These observations were consistent with those of a previous study [21], which revealed that the enhanced expression of MurA, MurG, and Ddl in a mutant at pH 3.5 contributed to peptidoglycan synthesis, maintaining cellular structural integrity in *Lactobacillus casei*Zhang. In addition, previous transcriptome analysis suggested that inducing peptidoglycan synthesis was a strategy that enhanced the ability of the cell wall to block H^+^ in *Bifidobacterium*[5]. In this study, the results of two-dimensional protein profiles and physiological tests also suggested that pre-stressing at pH 4.5 induced peptidoglycan synthesis in *B*. *longum*BBMN68, strengthening the cell wall in a lethal pH environment. This is the first study demonstrating a relationship between acid stress at lethal pH and peptidoglycan synthesis in *Bifidobacterium*.

**Effect on protecting against protein damage.** Faced with lethal pH acid stress, translation elongation factor (TurfB) and some aminoacyl-tRNA synthetases were specifically upregulated in *B*. *longum* BBMN68 cells that had been pre-stressed. Similar results were obtained in *L*. *casei* Zhang, *Streptococcus mutans*, and *Propioni bacterium fischeri*, in which the expression of TurfB was induced in a low pH environment [22–24]. Increasing the synthetic rate of TurfB increases the efficiency of aminoacyl-tRNA binding to TurfB, which helps protect proteins [22]. Therefore, in addition to an elongation function, TurfB also plays a role in protecting proteins from acid stress. This suggests that TurfB plays a role in the ASR in *Bifidobacterium*.

A previous study demonstrated that the proteolytic system of lactic acid bacteria is involved in more physiological and universal functions such as protein turnover, protein maturation, signal peptide processing, degradation of abnormal proteins, and inactivation of regulatory proteins [24]. In this study, the abundance of PepP, which is a member of the proteolytic system, was upregulated after the ASR. Similar findings have been reported for *L*. *casei* Zhang and *B*. *longum* biotype *longum*NCIMB8809 [6, 25]. The abundance of PepP was increased in MRS at pH 2.5 in *L*. *casei* Zhang [25]. The abundance of PepP was higher in *B*. *longum* biotype*longum*NCIMB8809 during growth at pH 4.8 compared with pH 7.0 [6]. These results might indicate that PepP plays a role in the ASR in *Bifidobacterium*.

The molecular chaperones GroEL, DnaJ1, and DnaK were upregulated after the ASR in *B*. *longum* BBMN68 cells without pre-stressing; however, no significant changes were detected after the ASR in BBMN68 *B*. *longum* cells with pre-stressing. These results suggested the possibility that protein damage was greater in the control-pH 3.5 group than in the pre-stressed-pH 3.5 group. In other words, pre-stressing improved the structural stability of proteins in *Bifidobacterium* in a lethal pH environment. These results are consistent with a previous study that indicated that the abundance of GroEL and DnaK in acid-resistant mutants was higher than in wild-type *L*. *casei* Zhang at pH 3.5 [23].

**Effect on the ability to maintain the intracellular pH.** The correctpH_in_ is crucial for bacterial growth. In a low pH environment, H^+^ enters the cell and decreases the pH_in_, resulting in damage to DNA and proteins. Therefore, the survival of bacteria in a low pH environment depends on their ability to maintain their pH_in_[15, 26]. In general, the main strategies to deal with acid stress in bacteria are discharging H^+^ via the proton pump, neutralizing H^+^ by producing alkaline metabolites, and expending H^+^ via biochemical reactions [6, 27, 28]. Compared with cells in the control group, the activity of H^+^-ATPase and the cellular NH_3_ content in the pre-stressed group was substantially higher (Fig. 4). These results suggested that pre-stressing strengthened the ability of *B*. *longum* BBMN68 to discharge H^+^ and neutralize H^+^. This explains why the ability of *B*. *longum* BBMN68 cells to maintain their pH_in_ was significantly greater with pre-stressing than without pre-stressing (Fig. 5).

## Conclusion

Based on the analysis of protein profiles and the verification of physiological data, this study yielded the following conclusions. Compared with the ATR, *Bifidobacterium* cells during the ASR had improved carbohydrate metabolism, H^+^-ATPase activity, and protein protection. Pre-stressing improved the ability of ATP production, NH_3_ production, protein protection, peptidoglycan synthesis, and pH_in_ maintenance in *Bifidobacterium* during the ASR. Our findings provide important insight into the effect of pre-stressing on the ability of *Bifidobacterium* to tolerate an acidic environment, and will aid the development of *Bifidobacterium* as an effective probiotic in food.

## Supporting Information

S1 File Contains the following files: **Table A.** Information on the proteins that changed abundance at low pH. **Fig. A.** Expression changes at the transcriptional level (line A) and the translational level (line B) during the ATR in *Bifidobacterium*. Black, up-regulated by ≥ two-fold; gray, down-regulated ≥ two-fold; white, no significant change.
(DOCX)
