# Supplementary material for: Effect of Pre-Stressing on the Acid-Stress Response in Bifidobacterium Revealed Using Proteomic and Physiological Approaches
Source: PLoS One. 2015 Feb 17;10(2):e0117702. doi: 10.1371/journal.pone.0117702 (PMC4331358; doi:10.1371/journal.pone.0117702)
Supplement: S1 File — Table A. Information on the proteins that changed abundance at low pH. Fig. A. Expression changes at the transcriptional level (line A) and the translational level (line B) during the ATR in Bifidobacterium. Black, up-regulated by ≥ two-fold; gray, down-regulated ≥ two-fold; white, no significant change. (DOCX) [file pone.0117702.s001.docx]

**Supplementary material 1**

**Table A Information of the proteins with changed abundance at low pH**

| Spot NO.^&^ | COG | Name | Function | Locus | GI | MW | *p*I | Protein score^△^ | Sequence coverage (%)^△△^ | Change fold＃ | | |
| --- | --- | --- | --- | --- | --- | --- | --- | --- | --- | --- | --- | --- |
|  |  |  |  |  |  |  |  |  |  | ATR* | ASR** | ASR'*** |
| ADD0 | C | AtpG | F_0_F_1_-type ATP synthase, γ-subunit | *BBMN68_1119* | GI:312133382 | 33902 | 5.65 | 127 | 32 | 1.6 | 2.84 | 2.31 |
| 1232 | C | Ppa | Inorganic pyrophosphatase | *BBMN68_1128* | GI:312133391 | 18440 | 4.42 | 157 | 62 | 1.26 | 0.46 | 0.45 |
| 1144 | C | NfnB2 | Nitroreductase | *BBMN68_1435* | GI:312133694 | 28418 | 5.15 | 57 | 22 | 1.86 | 0.74 | - |
| 870 | C | UgpQ | Glycerophosphoryl diester phosphodiesterase | *BBMN68_1512* | GI:312133771 | 40164 | 4.83 | 122 | 51 | 5.89 | 1.31 | 0.35 |
| 859 | C | PntA2 | NAD(P) transhydrogenase α- subunit | *BBMN68_597* | GI:311773831 | 39604 | 4.78 | 190 | 60 | - | 1.64 | + |
| 861 | C | AckA | Acetate kinase | *BBMN68_728* | GI:312132993 | 44273 | 5.47 | 148 | 42 | 1.13 | 1.24 | 2.33 |
| 838 | C | GalT1 | Galactose-1-phosphate uridylyltransferase | *BBMN68_977* | GI:312133240 | 47043 | 5.27 | 279 | 63 | 0.63 | 1.52 | 2.1 |
| 876 | C | DhaT | 1,3-propanediol dehydrogenase | *BBMN68_1706* | GI:312133965 | 41909 | 4.89 | 106 | 50 | 2.17 | - | 0.21 |
| 1046 | C | Tas1 | Aldo/keto reductase family oxidoreductase | *BBMN68_772* | GI:312133037 | 31939 | 5.02 | 56 | 5 | 1.1 | 0.53 | 0.16 |
| 724 | D | FtsZ | Cell division protein FtsZ | *BBMN68_165* | GI:312132440 | 41512 | 4.35 | 154 | 40 | 1.84 | 0.76 | 0.48 |
| 747 |  |  |  |  |  |  |  |  |  | 0.82 | 0.36 | 0.6 |
| 734 | D |  | Hypothetical protein | *BBMN68_168* | GI:311773087 | 50596 | 4.66 | 80 | 30 | 1.16 | 1.39 | 2.56 |
| 815 | D | FtsE | ATPase for cell division | *BBMN68_945* | GI:312133210 | 41855 | 4.78 | 132 | 40 | - | 2.35 | + |
| 802 | E | AspC | Aspartate aminotransferase | *BBMN68_1360* | GI:311772878 | 47383 | 5.34 | 157 | 41 | - | 0.97 | + |
| 1162 | E | HisA | Phosphoribosylformimino-5-aminoimidazole carboxamide ribonucleotide (ProFAR) isomerase | *BBMN68_185* | GI:312132460 | 25788 | 4.77 | 145 | 67 | 1.16 | - | 0.27 |
| 693 | E | PepP | Xaa-Pro aminopeptidase | *BBMN68_241* | GI:312132516 | 58484 | 4.84 | 104 | 40 | 0.32 | 1.11 | 3.76 |
| 1114 | E | HisG | ATP phosphoribosyltransferase | *BBMN68_464* | GI:312132735 | 30988 | 5.65 | 187 | 52 | 0.93 | 0.78 | - |
| 829 | E | ArgG | Argininosuccinate synthase | *BBMN68_809* | GI:311774067 | 45821 | 5.05 | 152 | 47 | - | 1.01 | + |
| 743 | E | Glna1 | Glutamine synthetase | *BBMN68_187* | GI:312132462 | 50040 | 4.84 | 137 | 33 | 0.31 | 0.82 | 4.56 |
| 855 | E | CsdB | Selenocysteine lyase | *BBMN68_609* | GI:312132874 | 45447 | 5.74 | 256 | 56 | 0.55 | 1.26 | 2.43 |
| 1056 | E | HisP1 | ABC-type amino acid transport system ATPase component | *BBMN68_61* | GI:311773852 | 30004 | 6.09 | 82 | 56 | 0.37 | 1.57 | 2.89 |
| 848 | E | AhsA2 | O-acetylhomoserine sulfhydrylase | *BBMN68_681* | GI:312132946 | 47571 | 5.1 | 208 | 68 | - | 2.3 | + |
| 1000 | EH | IlvC1 | Ketol-acid reductoisomerase | *BBMN68_1262* | GI:312133525 | 38707 | 5.10 | 177 | 46 | 2.61 | 0.55 | 0.67 |
| 1005 |  |  |  |  |  |  |  |  |  | 3.55 | 1.3 | 1.9 |
| 692 | F | PurA | Adenylosuccinate synthase | *BBMN68_1276* | GI:312133539 | 46839 | 5.4 | 188 | 42 | - | 3.85 | + |
| 1165 | F | Upp | Uracil phosphoribosyltransferase | *BBMN68_1514* | GI:312133773 | 23408 | 5.27 | 237 | 84 | 0.84 | 0.32 | 0.06 |
| 617 | F | Pnp | Polyribonucleotide nucleotidyltransferase | *BBMN68_1576* | GI:312133835 | 105483 | 5.01 | 107 | 19 | 0.28 | 0.61 | 2.25 |
| 752 | F | ThfS2 | Formyltetrahydrofolate synthetase | *BBMN68_1214* | GI:312133477 | 54309 | 5.04 | 191 | 37 | 0.54 | 1.31 | 2.3 |
| 1016 | FP | GppA2 | Exopolyphosphatase | *BBMN68_1776* | GI:312134035 | 35347 | 6.05 | 200 | 77 | 1.13 | 5.81 | 1.32 |
| 687 | G | Pgm | Phosphoglucomutase | *BBMN68_1663* | GI:311773216 | 60329 | 4.94 | 239 | 41 | 0.94 | 0.36 | 0.47 |
| 917 | G | MipB | Transaldolase | *BBMN68_407* | GI:311773634 | 39739 | 4.87 | 212 | 63 | 0.66 | 0.57 | 2.42 |
| 918 |  |  |  |  |  |  |  |  |  | 0.23 | 1.66 | 6.7 |
| 670 | G | Xfp | Phosphoketolase | *BBMN68_708* | GI:312132973 | 92683 | 5.06 | 118 | 21 | - | 3.63 | + |
| 707 |  |  |  |  |  |  |  |  |  | 0.71 | 3.18 | 3.85 |
| 725 |  |  |  |  |  |  |  |  |  | - | 1.14 | + |
| 731 |  |  |  |  |  |  |  |  |  | - | 2.91 | + |
| 742 |  |  |  |  |  |  |  |  |  | - | 1.54 | + |
| 748 |  |  |  |  |  |  |  |  |  | - | 1.28 | + |
| 843 |  |  |  |  |  |  |  |  |  | 0.78 | 3.32 | 1.62 |
| 850 |  |  |  |  |  |  |  |  |  | - | 2.45 | + |
| 864 |  |  |  |  |  |  |  |  |  | 1.5 | 1.03 | 0.43 |
| 878 |  |  |  |  |  |  |  |  |  | 0.42 | 2.57 | 3.39 |
| 980 |  |  |  |  |  |  |  |  |  | 0.6 | 2.62 | 2.46 |
| 1030 |  |  |  |  |  |  |  |  |  | - | 1.73 | + |
| 1269 |  |  |  |  |  |  |  |  |  | 0.53 | 3 | 2.86 |
| 833 |  |  |  |  |  |  |  |  |  | 0.63 | 2.33 | 1.66 |
| 816 |  |  |  |  |  |  |  |  |  | 0.55 | 2.8 | 1.96 |
| 824 | G | Eno | Enolase | *BBMN68_771* | GI:312133036 | 46600 | 4.7 | 224 | 60 | 0.99 | 0.77 | 2 |
| 809 | G | GalK | Galactokinase | *BBMN68_976* | GI:311772442 | 44560 | 4.93 | 140 | 42 | 2.19 | 0.69 | 0.47 |
| 812 | G | GlgC | ADP-glucose pyrophosphorylase | *BBMN68_606* | GI:312132871 | 45803 | 5.48 | 78 | 36 | 0.35 | 2.45 | 7.72 |
| 1055 | G | GlkA | Glucokinase | *BBMN68_1723* | GI:312133982 | 33257 | 5.06 | 108 | 41 | 0.83 | 0.2 | 0.28 |
| 1124 | G | RpiA | Ribose-5-phosphate isomerase A | *BBMN68_1657* | GI:312133916 | 25253 | 4.83 | 177 | 69 | 0.83 | 0.49 | 0.68 |
| 665 | G | SacA | Sucrose-6-phosphate hydrolase | *BBMN68_151* | GI:311772949 | 58339 | 4.87 | 170 | 36 | 0.66 | 0.33 | 0.58 |
| 774 | G | GapA | Glyceraldehyde-3-phosphate dehydrogenase | *BBMN68_254* | GI:311773463 | 37879 | 5.16 | 170 | 46 | 1.36 | 3.83 | 3.06 |
| 785 |  |  |  |  |  |  |  |  |  | - | 1.09 | + |
| 740 | G | Kdgk | Fructokinase | *BBMN68_230* | GI:312132505 | 59125 | 5.02 | 94 | 31 | 1.04 | 2.72 | 4.53 |
| 1214 | J | Efp | Translation elongation factor P | *BBMN68_111* | GI:311772575 | 20698 | 5.05 | 134 | 40 | 0.98 | - | 0.09 |
| 746 | J | GatA | Glutamyl-tRNA(Gln) amidotransferase A subunit | *BBMN68_1143* | GI:312133406 | 54150 | 4.96 | 119 | 40 | 0.76 | 0.14 | - |
| 732 | J | GlyS | Glycyl-tRNA synthetase class II | *BBMN68_163* | GI:311773043 | 56216 | 5.42 | 196 | 39 | 0.69 | 1.84 | 2.39 |
| 633 | J | ProS | Prolyl-tRNA synthetase | *BBMN68_1761* | GI:311773326 | 66227 | 4.84 | 284 | 42 | 0.26 | 0.75 | 2.71 |
| 890 | J | PheS | Phenylalanyl-tRNA synthetase alpha subunit | *BBMN68_818* | GI:312133083 | 38503 | 5.01 | 85 | 39 | 0.98 | 0.42 | 0.79 |
| 836 | J | TyrS | Tyrosyl-tRNA synthetase | *BBMN68_802* | GI:311774060 | 48412 | 5.08 | 73 | 29 | 0.78 | 0.6 | - |
| 794 |  |  |  |  |  |  |  |  |  | 0.84 | 3.08 | 3.95 |
| 1177 | J | Rpld | Ribosomal protein L4 | *BBMN68_1616* | GI:312133875 | 23490 | 9.76 | 113 | 48 | 1.8 | 15.8 | 1.17 |
| 674 | J | FusA4 | Elongation factor G | *BBMN68_846* | GI:312133111 | 78429 | 4.83 | 256 | 40 | 1.15 | 0.45 | 0.87 |
| 671 | J | MetG | Methionyl-tRNA synthetase | *BBMN68_1466* | GI:312133725 | 66785 | 4.94 | 108 | 22 | 1.91 | 0.38 | 0.38 |
| 892 | J | Gcd14 | tRNA(1-methyladenosine) methyltransferase | *BBMN68_540* | GI:312132807 | 39998 | 6.05 | 114 | 40 | 0.74 | 3.24 | 4.47 |
| 796 | JE | TufB | Translation elongation factor | *BBMN68_845* | GI:311774107 | 43908 | 4.88 | 221 | 61 | 1.05 | 1.18 | 3.11 |
| 1254 | K | GreA | Transcription elongation factor | *BBMN68_764* | GI:312133029 | 17155 | 4.82 | 90 | 69 | 3.52 | 0.23 | 0.15 |
| 1179 | L | Ssb2 | Single-stranded DNA-binding protein | *BBMN68_1155* | GI:312133418 | 22646 | 4.9 | 123 | 53 | 0.99 | - | - |
| 726 | L | RecN | ATPase involved in DNA repair | *BBMN68_57* | GI:311773464 | 53498 | 5.26 | 183 | 31 | 0.62 | 1.3 | 2.03 |
| 1229 | L | Dps | Starvation-inducible DNA-binding protein | *BBMN68_1349* | GI:312133610 | 17797 | 4.57 | 151 | 61 | 1.55 | 0.77 | 1.09 |
| 738 | M | MurC | UDP-N-acetylmuramate--L-alanine ligase | *BBMN68_210* | GI:311773413 | 53522 | 5.35 | 124 | 60 | 0.42 | 1.44 | 2.77 |
| 934 | M | GalE1 | UDP-glucose 4-epimerase | *BBMN68_1676* | GI:312133935 | 37260 | 5.27 | 95 | 46 | 0.58 | 1.61 | 2.49 |
| 986 | M | CbaH | Conjugated bile acid hydrolase | *BBMN68_536* | GI:312132803 | 35123 | 4.66 | 147 | 57 | 2.63 | 0.45 | 0.8 |
| 998 |  |  |  |  |  |  |  |  |  | 2.44 | 0.72 | 0.41 |
| 795 | N | MalK | ABC-type sugar transport systems, ATPase components | *BBMN68_1403* | GI:312133662 | 40702 | 5.85 | 122 | 43 | 0.49 | 1.67 | 5.57 |
| 800 |  |  |  |  |  |  |  |  |  | 1.62 | 4.82 | 3.36 |
| 905 |  |  | Hypothetical protein BLD_0908 | *BBMN68_912* | GI:312133177 | 43245 | 5.12 | 127 | 37 | 1.45 | 2.55 | 0.95 |
| 1119 | O | GrpE | Molecular chaperone GrpE ( heat shock protein ) | *BBMN68_1251* | GI:312133514 | 23555 | 4.54 | 128 | 52 | 2.5 | 0.86 | 0.47 |
| 639 | O | DnaK | Chaperone protein DnaK | *BBMN68_1252* | GI:311772760 | 67356 | 4.72 | 126 | 50 | 0.69 | 0.63 | 3.01 |
| 647 |  |  |  |  |  |  |  |  |  | - | 3.58 | + |
| 817 | O | DnaJ1 | Chaperone protein | *BBMN68_410* | GI:312132681 | 40567 | 6.07 | 167 | 58 | 0.77 | 2.15 | 2.53 |
| 691 | O | GroL | Chaperonin GroEL | *BBMN68_44* | GI:312132319 | 56802 | 4.72 | 137 | 35 | 0.96 | 1.8 | 4.2 |
| 993 | T | UspA1 | Universal stress protein | *BBMN68_51* | GI:312132326 | 34522 | 5.39 | 84 | 30 | 3.34 | 0.8 | 0.31 |

- &, the spot NO. was consistent with the number marked SDS-PAGE in Figure ２.
- △, MASCOT protein score from mass spectrometry (MS).
- △△, Percentage of amino acids in reference proteins covered by matching peptides from MS.

＃the ratio of the abundance of protein of different groups

* = the abundance of protein in Induction Group / the abundance of protein in Control Group

** = the abundance of protein in Control-pH3.5 Group / the abundance of protein in Control Group

*** = the abundance of protein in Induction-pH3.5 Group / the abundance of protein in Induction Group

- means the spot wasn't detectable after the treatment(in the numerator groups), + means the spot wasn't detectable before the treatment(in the denominator groups).

**Supplementary material 1.**


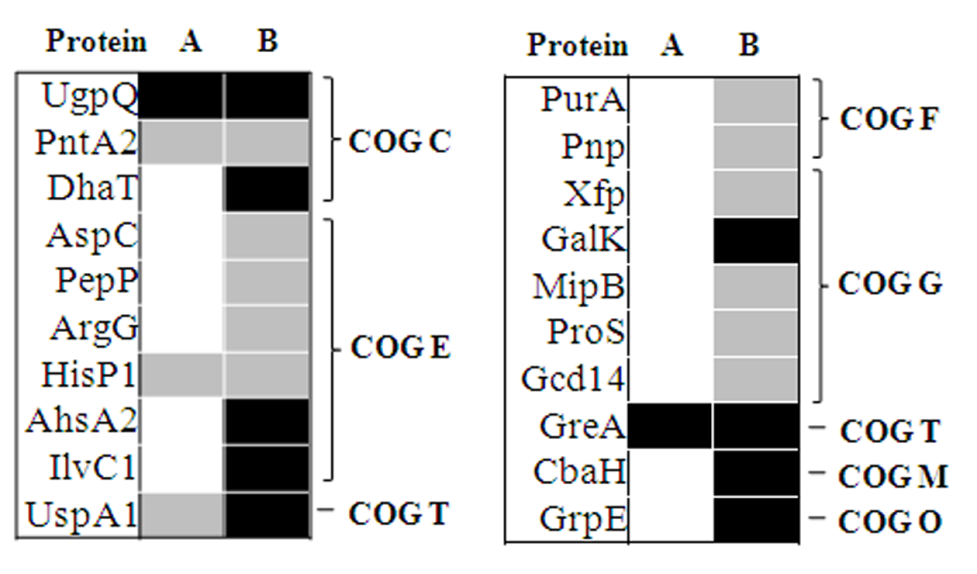


**Figure A. Change of gene expression at transcriptional level (line A)and translational level(line B) during ATR.** Black, up-regulated by ≥2- fold. Grey, down-regulated ≥2- fold. White, no significant change.
